# Expression Profiling of Blood microRNAs 885, 361, and 17 in the Patients with the Parkinson’s disease: Integrating Interaction Data to Uncover the Possible Triggering Age-Related Mechanisms

**DOI:** 10.1038/s41598-019-50256-3

**Published:** 2019-09-24

**Authors:** Molood Behbahanipour, Maryam Peymani, Mehri Salari, Motahare-Sadat Hashemi, Mohammad Hossein Nasr-Esfahani, Kamran Ghaedi

**Affiliations:** 1 Department of Cell and Molecular Biology and Microbiology, Faculty of Biological Science and Technology, University of Isfahan, Isfahan, Iran; 2 Department of Biology, Faculty of Basic Sciences, Shahrekord Branch, Islamic Azad University, Shahrekord, Iran; 3 Department of Cellular Biotechnology, Cell Science Research Center, Royan Institute for Biotechnology, ACECR, Isfahan, Iran; 4 Functional Neurosurgery Research Center, Shohada Tajrish Neurosurgical Center of Excellence, Shahid Beheshti University of Medical Sciences, Tehran, Iran

**Keywords:** Parkinson's disease, Predictive markers, Predictive markers, Predictive markers, Parkinson's disease

## Abstract

MicroRNAs (miRNAs) have been reported to contribute to the pathophysiology of the Parkinson’s disease (PD), an age related-neurodegenerative disorder. The aim of present study was to compare the expression profiles of a new set of candidate miRNAs related to aging and cellular senescence in peripheral blood mononuclear cells (PBMCs) obtained from the PD patients with healthy controls and then in the early and advanced stages of the PD patients with their controls to clarify whether their expression was correlated with the disease severity. We have also proposed a consensus-based strategy to interpret the miRNAs expression data to gain a better insight into the molecular regulatory alterations during the incidence of PD. We evaluated the miRNA expression levels in the PBMCs obtained from 36 patients with PD and 16 healthy controls by the reverse transcription-quantitative real-time PCR and their performance to discriminate the PD patients from the healthy subjects assessed using the receiver operating characteristic curve analysis. Also, we applied our consensus and integration approach to construct a deregulated miRNA-based network in PD with the respective targets and transcription factors, and the enriched gene ontology and pathways using the enrichment analysis approach were obtained. There was a significant overexpression of miR-885 and miR-17 and the downregulation of miR-361 in the PD patients compared to the controls. The blood expression of miR-885 and miR-17 tended to increase along with the disease severity. On the other hand, the lower levels of miR-361 in the early stages of the PD patients, as compared to controls, and its higher levels in the advanced stages of PD patients, as compared to the early stages of the PD patients, were observed. Combination of all three miRNAs showed an appropriate value of AUC (0.985) to discriminate the PD patients from the healthy subjects. Also, the deregulated miRNAs were linked to the known PD pathways and the candidate related target genes were presented. We revealed 3 candidate biomarkers related to aging and cellular senescence for the first time in the patients with PD. Our *in-silico* analysis identified candidate target genes and TFs, including those related to neurodegeneration and PD. Overall, our findings provided novel insights into the probable age-regulatory mechanisms underlying PD and a rationale to further clarify the role of the identified miRNAs in the PD pathogenesis.

## Introduction

Parkinson’s disease (PD) is the second most common age-related neurodegenerative disorder after Alzheimer’s disease, affecting the movement abilities. PD incidence is rare before the age of 50, but its occurrence rises from 5 to 10 folds in the age range of 60 to 90^1^. Despite extensive studies, PD etiology and pathology have still remained largely unknown. Hence, a causal therapy for PD is currently not available, and its clinical diagnosis is mainly based on the presence of characteristic motor symptoms such as bradykinesia, muscle rigidity, resting tremor, and postural and gait impairment when neurodegeneration in *substantia nigra* (SN) *pars compacta* reaches the thresholds value of around 60%^2,3^.

Due to the heterogeneous nature of PD, a combination of aging, genetic and non-genetic risk factors are involved^4^; the current approach has been aimed to understand its underlying mechanisms at different levels of regulation. In this regard, microRNAs (miRNAs), a class of non-coding RNAs, have been reported to play an important role in PD^5^. Mature miRNAs are single-stranded RNAs of approximately 21–25 nucleotides in length, which are capable of post-transcriptionally regulating their target messenger RNAs (mRNAs) by mainly binding to the 3′ untranslated regions (3′UTR), leading to mRNA degradation or translational repression. A single 3′UTR may contain binding sites for various miRNAs or multiple sites for a single miRNA, representing the complex post-transcriptional regulation of the gene expression by miRNAs partaking in diverse cellular functions including cell proliferation, cell death, growth pathways, differentiation and immune system, which can be involved in the molecular mechanisms of various diseases. In human, there is at least one conserved miRNA-binding site in more than 60% of the protein-coding genes. Moreover, numerous non-conserved miRNA-binding sites are also present; therefore, most protein-coding genes may be under miRNA regulation^6,7^. MiRNAs also exist in a highly stable form in the peripheral blood and their expression profiles can be easily measurable, making them ideal biomarkers. In this regard, recently, a number of studies have investigated the expression profiles of several PD candidate miRNAs in the peripheral blood mononuclear cells (PBMCs) obtained from the PD patients, as compared to the healthy controls^8–11^.

Aging is recognized to be the greatest risk factor for developing idiopathic PD^1^. However, the specific age-related factors that trigger PD in the elderly are not still known. Therefore in this study, in order to better understand the possible contributing factors involved in these mechanisms, the expression of a set of candidate miRNAs including miR-17, miR-361 and miR-885 has been profiled in the PBMCs obtained from the PD patients and healthy controls; it has been previously demonstrated that as miRNAs are involved in aging and cellular senescence^12–16^. These miRNAs have also shown differential expression in the miRNA expression microarray dataset [GSE16658: consisted of 19 PD patients and 13 controls in the GEO database (https://www.ncbi.nlm.nih.gov/geo/)] in the PBMCs of the PD patients and controls^11^. Thus, in this study, we have not only confirmed the differential expression of these miRNAs in the PD patients compared to the healthy controls, but also presented them as a novel candidate set of aging and cellular senescence and evaluated associations of their PBMCs levels in relation to the disease severity via their expression profiling in both the early and advanced stages of the PD patients with their controls. Furthermore, miRNAs, as the regulatory molecules, are also under regulation, the most important of which is the miRNA-transcription factor (TF) regulatory network. In order to reach a more comprehensive view of the functional roles of these miRNAs in the PD pathology, we performed an enrichment analysis on the putative target genes and the TFs of these miRNAs.

## Methods

### Patients

This study was approved by the local ethics committee of Royan Institute (IR.ACECR.ROYAN.REC.1397.10). Meanwhile, all participants signed the written informed consent. All methods were performed in accordance with the relevant guidelines. Thirty-six patients with PD were recruited from Al-Zahra Hospital, Isfahan, Iran. The clinical diagnosis of PD was confirmed by a certified movement disorder neurologist, according to the UK Brain Bank criteria^17^. Subjects with other neurological diseases, atypical parkinsonism, drug-induced secondary parkinsonism, cognitive impairment, diabetes, renal dysfunction, infection, tumor, cardiovascular and cerebrovascular diseases were excluded. Exclusion criteria for the patients with cognitive impairment were those with Montreal Cognitive Assessment (MoCA) score less than 26^18^. The severity and stage of the PD symptoms were evaluated using the modified Hoehn and Yahr stage (HY)^19^. Thirteen patients with the unilateral motor impairment only (HY-1 stage) and fifteen patients with the bilateral or midline involvement in the absence of the impairment of balance (HY-2 stage) were classified as the early stage PD. In contrast, five patients with the postural reflexes impairment (HY-3 stage) and three patients with severe disability, who still were able to walk or stand unassisted (HY-4 stage), were classified as the advanced stage PD. In addition, we enrolled sixteen volunteer control subjects with no history of neurological or psychiatric diseases from spouses, unrelated caregivers and outpatient clinics.

### PBMCs isolation

Five mL of the whole blood was collected in an EDTA-containing tube from all participants and processed within 6 hours of blood collection. PBMCs were isolated using centrifugation through a density gradient medium by Lymphodex (Inno-Train Diagnostik GmbH, Kronberg, Germany), according to the instructions of manufacturer. Cells were washed twice with phosphate buffered saline (PBS) (Gibco, Thermo Fisher Scientific, Waltham, MA, USA), counted and resuspended at the desired concentration.

### RNA extraction and quality control

Total RNAs, including small RNAs, were extracted from PBMCs using the TRIzol reagent (Invitrogen, Carlsbad, CA, USA), according to the manufacturer’s protocol. We determined the RNA quality and quantity using a NanoDrop 2000/2000c spectrophotometer (Thermo Fisher Scientific, Waltham, MA, USA) and the 260/280 nm absorbance ratio was evaluated. All samples showed an absorbance ratio between 1.8 and 2. In addition, to eliminate any potential genomic DNA (gDNA) contamination, the total RNA samples were treated with RNase-free DNase I (Fermentas, Thermo Fischer Scientific, Waltham, MA, USA), according to the manufacturer’s instructions.

### Reverse transcription and the quantitative real-time PCR

Reverse transcription (RT) reaction was performed on 500 ng of the total RNA using the universal cDNA synthesis kit II (Exiqon, Vedbaek, Denmark) in a 10 μL reaction. The thermal cycling parameters were 60 minutes at 42 °C, 5 minutes at 95 °C for the heat-inactivation of the reverse transcriptase, and immediate cooling at 4 °C.

Subsequently, the real-time quantitative PCR (RT-qPCR) of hsa-miR-17-5p, hsa-miR-361-5p, hsa-miR-885-5p, and the candidate normalizer hsa-miR-191-5p was performed in triplicate with the specific primers on the Step One Plus Real-Time PCR thermal cycler (Applied Biosystems, Foster City, CA, USA) using SYBR Green Master Mix: SYBR Premix Ex *Taq*II (TaKaRa, Tokyo, Japan) and microRNA LNA™ primer sets (Exiqon, Vedbaek, Denmark); in accordance with the manufacturer’s instructions. The thermal cycling parameters were 10 minutes at 95 °C; this was followed by 40 cycles of 10 seconds at 95 °C and 1 minute at 60 °C.

Relative expression levels were calculated using the comparative C_T_ method with hsa-miR-191-5p as the normalizing control.

### Statistical analysis

Data are presented as the means ± standard error of the mean (SEM). The Shapiro-Wilk test was used to evaluate whether each variable followed the normal distribution; according to its results, normality was not proved. So, for the group-wise comparisons, the Mann-Whitney U test for 2 groups and the Kruskal-Wallis H test for n groups were used.

Receiver operating characteristic (ROC) curve analysis was used for each miRNA and using binomial logistic regression to combine miRNAs. The statistical analysis was performed using the IBM SPSS software, version 23.0 (SPSS Inc., Chicago, IL, USA) and GraphPad Prism 6.0 (GraphPad Software, USA). Significant differences were defined as *p* < 0.05.

### microRNA target analysis

Five miRNA target databases were used, including TargetScan (release 7.2), microT-CDS (release 5.0) and miRDB (release 5.0); these consisted of computationally predicted miRNA targets^20–22^. On the other hand, miRTarBase (release 7.0) and TarBase (release 8.0) contained the experimentally validated miRNA targets^23,24^. Within them, we only retrieved the common predicted targets for miR-885-5p, miR-17-5p and miR-361-5p from TargetScan with a total context ++ score <−0.2, microT-CDS with an miTG score ≥0.8, and miRDB with a score >80. These strict thresholds were chosen to obtain a high-confidence list of the candidate miRNA-target interactions and reduce the number of false positives. We also extracted their validated targets with strong evidence (reporter assay, western blot, qPCR) from miRTarBase and TarBase.

### Transcription factor-microRNA analysis

Transcription factors (TFs) regulating the transcription of miR-885, miR-17 and miR-361 were obtained from the TransmiR (release 2.0) database^25^. Within them, we only retrieved validated and more stringent evidence-level TF-miRNA interactions.

### Regulatory network construction

We constructed a miRNA-based network which included miR-885-5p, miR-17-5p and miR-361-5p, their targets and TFs. The network was generated and visualized using the Cytoscape (version 3.4.0) software^26^. In addition, TFs and miRNAs involved in the feedback loops (FBLs), as well as the confidence level of their regulation based on TransmiR, were depicted in a subnetwork. Due to miRNA-target interactions, we also assumed that all miRNAs repressed their targets.

### Functional enrichment and pathway analysis

In order to explore gene ontology (GO) biological processes, molecular function and cellular components predominantly shown in the miRNA-based network, we performed a functional annotation analysis using Enrichr (a comprehensive gene set enrichment analysis tool)^27^; the significantly enriched gene ontology terms (adjusted *p*-value < 0.05) were summarized using the REVIGO web server by removing the redundant GO terms and visualizing the remaining terms based on semantic similarity^28^. For the Go biological process, a 0.5 similarity was allowed (small list), while a 0.7 one was considered for the molecular function and cellular components (medium list). Moreover, significantly enriched PANTHER pathways^29^ (adjusted *p*-value < 0.001) were extracted from Enrichr and plotted using Microsoft Excel.

### Ethics approval and consent to participate

Approval from the local ethics committee of Royan Institute (IR.ACECR.ROYAN.REC.1397.10) was obtained; all participants signed the written informed consent prior to the related studies.

## Results

### Characteristics of the subjects

The clinical and demographic characteristics of the PD patients and healthy subjects are summarized in Table 1. Statistical analyses showed no significant differences in terms of gender and age between the groups.

**Table 1:** Demographic and clinical data of the PD patients and the control group.

	Controls	PD	Early stage PD		Advanced stage PD		*p-*Value
N	16	36	Hoehn & Yahr stage I	Hoehn & Yahr stage II	Hoehn & Yahr stage III	Hoehn & Yahr stage IV	—
			13	15	5	3	
Age (Year)	62.5 ± 12.4	61.3 ± 11.4	60.9 ± 10.6		62.8 ± 14.6		0.741^a^ 0.879^b^
F/M	5/11	11/25	8/20		3/5		0.960^c^ 0.889^d^
Disease Duration, (month)	—	69.6 ± 67.2	58.8 ± 57.8		111 ± 88.5		0.232^e^

Data are presented as mean ± SD. ^a^*p*-value was calculated using a two-tailed Student’s *t* test. ^b^*p*-value.

was calculated using one-way ANOVA. ^c,d^*p*-values were calculated using chi-square test. ^e^*p*-value.

was calculated using the Mann-Whitney test. As indicated, there was no significant difference between the controls and the PD patients based on the age and sexuality. PD stands for the Parkinson’s disease. F and M represent female and male, respectively.

### microRNAs expression in the PD patients and controls

**Figure 1:**
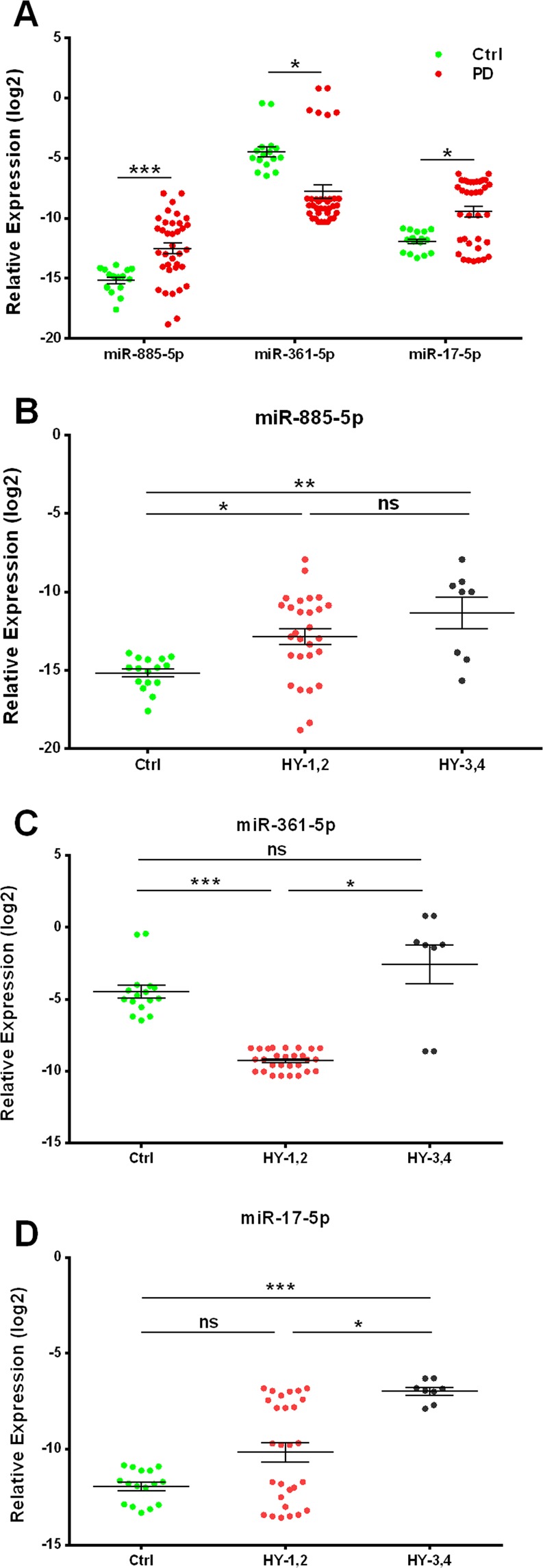
Assessment of the relative expression levels of miR-885-5p, miR-361-5p and miR-17-5p in the PBMCs of: (**A**) control subjects and PD patients. (**B**–**D**) Control subjects and early (HY-1,2) and advanced (HY-3,4) stage PD patients based on Hoehn & Yahr stages. Data are presented as the mean ± SEM. Differences were analyzed by the Mann-Whitney U test (**A**) and the Kruskal-Wallis H test (**B**–**D**) (***p ≤ 0.001, **p ≤ 0.01, *p ≤ 0.05, and ns *p* > 0.05). ns stands for the non-significance.

Quantitative analysis of the miRNA expression using RT-qPCR revealed that miR-885-5p and miR-17-5p expressions were significantly overexpressed, while the miR-361-5p expression was significantly reduced in the PBMCs of the PD patients, as compared to the controls (Fig. 1A). We also performed an exploratory expression analysis on the early and advanced stages in the PD patients (based on H & Y scale), as compared to the healthy controls. miR-885-5p demonstrated significant overexpression in both early and advanced stage patients, as compared to the control (Fig. 1B). On the other hand, miR-17-5p was significantly overexpressed in the advanced stage patients vs. healthy subjects and early-stage patients (Fig. 1D). Meanwhile, miR-361-5p revealed a significant reduction in the early-stage patients, as compared to the control group and significant overexpression in the advanced stage patients, in contrast to early-stage patients (Fig. 1C). In this regard, the mean difference (95% CI) for each miRNA within each group is represented (Table 2).

**Table 2:** Results of the miRNA expression analysis.

	miRNA	Mean difference (95% CI)	*p*-value
PD vs. controls	miR-885-5p	2.58 (1.37, 3.79)	<0.001
	miR-361-5p	−3.31 (−4.93, −1.7)	0.036
	miR-17-5p	2.48 (1.28, 3.68)	0.034
Early stage PD patients vs. controls	miR-885-5p	2.25 (0.15, 4.36)	0.034
	miR-361-5p	−4.95 (−7.71, −2.19)	<0.001
	miR-17-5p	1.72 (−0.35, 3.79)	0.116
Advanced stage PD patients vs. controls	miR-885-5p	3.67 (0.79, 6.55)	0.009
	miR-361-5p	1.75 (−1.60, 5.10)	0.332
	miR-17-5p	4.93 (2.25, 7.62)	<0.001
Advanced stage PD patients vs. Early stage PD patients	miR-885-5p	1.41 (−1.27, 4.10)	0.416
	miR-361-5p	6.70 (3.06, 10.34)	0.023
	miR-17-5p	3.22 (0.45, 5.98)	0.021

Abbreviations: CI = confidence interval; miRNA = microRNA; PD = Parkinson’s disease. The results are referred to the expression data elaborated using a reference gene miR-191-5p.

### microRNA as the predictive biomarkers

**Figure 2:**
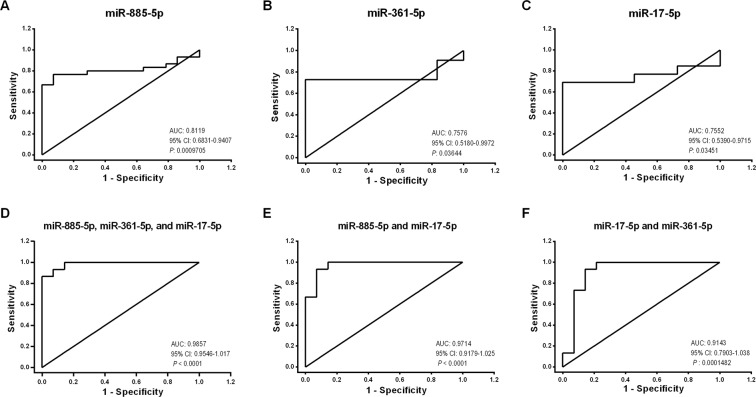
Receiver operating characteristic (ROC) curve analysis for: (**A**–**C**) miR-885-5p, miR-361-5p, and miR-17-5p in the PD patients versus the healthy controls. (**D**,**E**) combined microRNAs using binomial logistic regression in the PD patients versus the healthy controls.

Receiver operating characteristic (ROC) analysis, which is defined as a sensitivity plotted against 1-specificity, was performed to assess the performance of significantly differentially expressed miR-885-5p, miR-361-5p and miR-17-5p to discriminate the PD patients from the healthy subjects (Fig. 2A–C). In this regard, miR-885-5p, miR-361-5p and miR-17-5p showed a fair separation between the PD and healthy controls samples with an area AUC of 0.811, 0.757 and 0.755, respectively.

We then applied the binomial logistic regression to identify the best discriminating combinations of miRNAs (Fig. 2D–F) in which the combination of all three miRNAs showed an appropriate value of AUC (0.985), as compared to the best individual miRNA (miR-885-5p, AUC: 0.811) and the best pair of miRNAs (miR-885-5p and miR-17-5p, AUC: 0.971) (Fig. 2D–F). Notably, the value of AUC for the combination of miR-885-5p and miR-361-5p was not satisfying (AUC: 0.638) and significant (*p* = 0.2057) (Supplemental Fig. 1).

### Interaction analysis of miRNAs-target mRNAs

We then determined, predicted and validated the targets of three miRNAs. In this context, we found 126 predicted miRNA-target pairs via common-target screening with strict thresholds in TargetScan^20^, microT-CDS^21^ and miRDB^22^. Additionally, we extracted 133 validated miRNA-target pairs with strong validation methods from miRTarBase^23^ and TarBase^24^. There were some overlaps between the predicted and validated miRNA-target pairs. Hence, totally, we identified 247 miRNA-target interactions.

### Transcription Factor-miRNA regulation

We explored transcription factors (TFs) regulating the 3 miRNAs from TransmiR^25^. By using TransmiR, an experimentally supported TF-miRNA regulatory relationship database, 94 TF-miRNA interactions were identified. Among them, 12 TFs were part of the feedback loops (FBLs) with miRNAs.

### Construction of the miRNA-based regulatory network

**Figure 3:**
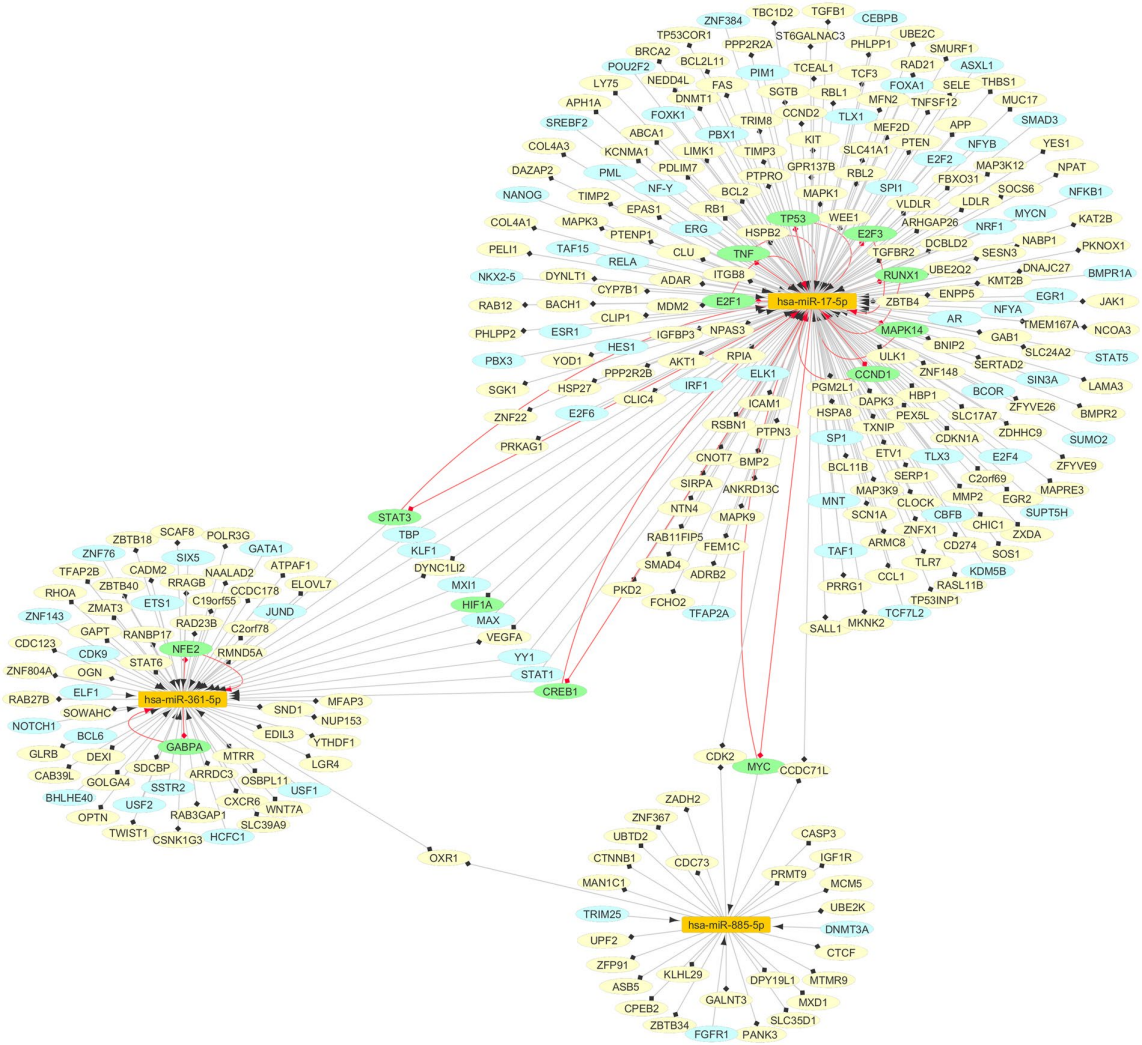
The miRNA-based network dysregulated in the PD. The orange nodes represent miRNAs, the yellow nodes represent the target genes, and the blue ones show the transcription factors (TFs); on the other hand, the green ones represent molecules that are TFs and also, the target genes. The red edges show the TF-miRNA feedback loops (FBLs).

**Figure 4:**
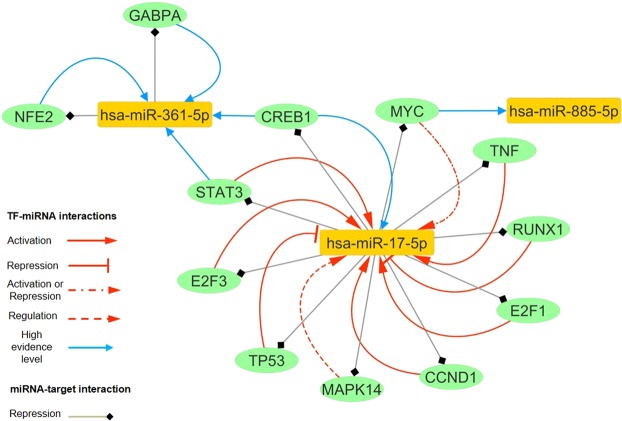
The nodes and edges involved in the feedback loops of the miRNA-based network. Orange nodes represent miRNAs, and the green ones show the molecules that are TFs and also, the target genes. Confidence level of TF-miRNA regulations is based on the TransmiR database. Regarding miRNA-target interactions, we also assumed that all miRNAs repressed their targets.

Further, we constructed a regulatory network based on 3 miRNAs, their TFs and target genes using the data obtained from the previous steps (Fig. 3). Our miRNA-based regulatory network consisted of 317 nodes (3 miRNAs, 84 TFs and 242 miRNA targets, while 12 TFs were also miRNA targets), and 341 directed edges (247 miRNA-target pairs and 94 TF-miRNA pairs). Overall, this network depicted 3 potential layers of the complex regulatory interactions based on 3 miRNAs, which could provide insight into the vast dysregulated network in PD. Furthermore, TFs were part of the FBLs with miRNAs, as illustrated in the subnetwork (Fig. 4).

### Functional enrichment and pathway analysis

**Figure 5:**
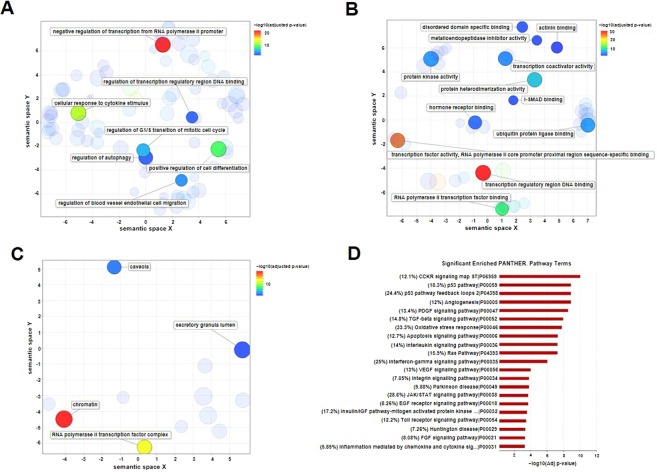
Gene Ontology (GO) and pathway enrichment analysis. (**A**–**C**) GO enrichment analysis was summarized and visualized as a scatter plot using REVIGO^28^. Scatter plot was generated in REVIGO by clustering the significant (adjusted *p*-value < 0.05) GO terms remaining after redundancy reduction for the biological processes (**A**), molecular functions (**B**) and cellular components (**C**) in a two-dimensional space (2D space), according to semantic similarities. The bubble color indicates -log_10_ (adjusted *p*-value) according to the legend. Bubble size refers to the frequency of the GO term in the underlying *Homo sapiens* database. Cluster representatives with the lowest dispensability value are labelled. (**D**) The bar plot shows −log_10_ (adjusted *p*-value) of the significantly enriched PANTHER pathways (adjusted *p*-value < 0.001) for all nodes of the microRNA-based network. Fold enrichment for each pathway is shown in parentheses as the percentage.

We performed a functional enrichment analysis for the Gene Ontology (GO) terms regarding the TFs and targets of the miRNA-based network using Enrichr^27^ to obtain a comprehensive view of the processes dysregulated during PD. In this context, significant enriched GO terms (adjusted *p*-value < 0.05) were summarized and visualized using REVIGO^28^ (Fig. 5A–C). Visualization of these results in the “biological processes” ontology pointed to the negative regulation of transcription from RNA polymerase II promoter, the cellular response to cytokine stimulus, the regulation of the transcription regulatory region DNA binding, the regulation of the blood vessel endothelial cell migration, the regulation of the G1/S transition of mitotic cell cycle, the regulation of autophagy, and the positive regulation of cell differentiation (Fig. 5A). Regarding “molecular functions” involving the transcription factor activity, transcription coactivator activity, protein kinase activity, metalloendopeptidase inhibitor activity, transcription regulatory region DNA binding, RNA polymerase II transcription factor binding, I-SMAD binding, disordered domain specific binding, actinin binding, protein heterodimerization activity, hormone receptor binding and ubiquitin protein ligase binding can be mentioned (Fig. 5B). These results indicated that three miRNAs were involved in the fundamental biological processes and molecular functions of PD pathophysiology. Furthermore, based on the analysis of terms in the “cellular component” ontology enriched for chromatin, caveola (including membrane raft) (Fig. 5C) were ones of the cellular components affected by PD.

Finally, significantly enriched (adjusted *p*-value < 0.001) PANTHER^29^ pathways for the miRNA-based network nodes indicated the nodes in the network were part of the potential pathways associated with PD, including p53 signaling pathways, TGF-β signaling pathway, oxidative stress response, and immunological pathways such as the response to interleukin and interferon-gamma signaling pathway (Fig. 5D). It should be noted that dopaminergic pathway was not identified through this analysis.

## Discussion

Aging is the greatest risk factor for PD^30^; however, the association between the molecular/cellular processes involved in physiologic aging and PD pathogenesis is still unclear. Therefore, focusing on aging regulators may provide more insight into the underlying PD pathophysiology that can be used for the future therapeutics or diagnostic studies.

In this study, we investigated differential expression (DE) of three miRNAs involved in aging and cellular senescence in the PBMCs obtained from the PD patients as the accessible tissue undergoing the inflammatory processes and cell death during the PD pathogenesis^31^. Then, logistic regression was used to develop a combined miRNA panel for a more specific and sensitive discrimination. Finally, a miRNAs-based network was created, and the potentially TF-miRNA feedback loops (FBLs) were determined. Based on this network, we were able to identify the gene ontology and pathways involved in PD.

In 2015, Prajapati *et al*. demonstrated the significant overexpression of miR-17 and eight other miRNAs in the TNF-α-treated SH-SY5Y cells (TNF: Tumor necrosis factor)^32^. In our miRNA-based network, miR-17 formed an FBL with TNF-α, which could transcriptionally activate it. It was visible on the network; miR-17 also downregulated E2F1 as a regulator of G1/S transition^33^ and WEE1 that is involved in G_2_/M transition^34^. Furthermore, a set of pro-proliferative and anti-proliferative target genes including CCND1, CDKN1A (p21), PTEN, BCL2L11 (BIM), RB1, RBL1 (p107) and RBL2 (p130) were shown to be suppressed by miR-17.

In 2010, Hackl *et al*. found the significant decrease of miR-17 in four different replicative cell aging and three organismal aging models, suggesting the use of miR-17 as a cellular aging biomarker. They also suggested the decreased miR-17 levels were associated with down regulation of E2F family members’ levels and the increased p53 activity in senescence^12,35,36^. As, E2F family members transcriptionally activate miR-17, while p53 represses it^37,38^. Notably, the same reduced expression pattern of miR-17 has been shown in PBMCs derived from the aging persons^13^. We know that lifelong exposure to exogenous and endogenous stressors can alter normal signaling mechanisms under pathological condition. For instance, deregulation of the E2F/Rb pathway, such as aberrant pRb expression and active E2F, has been detected in PD^39,40^. E2F/Rb pathway is critical for the cell-cycle regulation, in which pRb phosphorylation releases E2F family members to activate the expression of the genes involved in the regulation of DNA synthesis, cell cycle progression and apoptosis^41^. Interestingly, based on our miRNA-based network, miR-17 targets RBL1, RBL2 and RB1, which may result in the E2Fs activation. Also, miR-17 forms FBL with the activator E2Fs (E2F1 and E2F3), in which the activating effects of E2F1 and E2F3 probably overcome the inhibitory effect of miR-17 during the PD progression. We also found other transcription factors of miR-17, such as STAT3, CCND1, RELA, AR, ESR1, NANOG, SMAD3 and TFAP2A as its activators, and SPI1, MAX, MXI1, CEBPB, RUNX1 as its repressors. However, given the significant increased miR-17 levels in the advanced stage patients vs. the control and early stage patients in our study, it seems the miR-17 expression levels have been possibly regulated by the activator TFs during PD progression and further studies would be needed to confirm this.

Beside miR-17, the decreased miR-361 plasma levels were demonstrated during aging^14^. Chmielarz *et al*. (2017) also found the significant reduction of the miR-361 expression in the micro-dissected DA neurons while comparing old and young mice^15^. In our study, the expression levels of miR-361 were significantly reduced in the early stage patients in contrast to the control, and it was overexpressed in the advanced vs. early stage patients. Based on our miRNA-based network, miR-361 targets STAT6, a multifunctional cytokine that plays important roles through the modification of cell-specific differentiation, cell growth, gene regulation and induction of resistance to apoptosis^42,43^. Another target of miR-361 is GABPA (Nuclear Respiratory Factor 2), a transcription factor regulating the genes expression involved in the mitochondrial function^44,45^, immune response^46^, cell cycle progression^47–49^, cell survival^50^, hematopoietic stem and progenitor cells, and myeloid differentiation^51,52^. According to the network, GABPA also forms an FBL with miR-361, whose effects on each other during PD progression require further study. miR-361 also targets *OPTN*, a gene involved in protein trafficking^53^, the maintenance of the Golgi apparatus^54^, mediation of the autophagic flux, cell division, and NF-kB pathway. The increased midbrain OPTN expression with rotenone exposure in the pre-clinical and end-stage PD models has been reported^55^. Furthermore, Osawa *et al*. (2011) demonstrated the presence of optineurin-positive structures in various neurodegenerative diseases including PD^56^. However, the exact role of this OPTN alteration and how its levels are affected by miR-361 in the PD pathogenesis need to be clarified. Another target of miR-361, ZMAT3 (WIG1), has a stress-related function, which is a direct p53 target that is also a regulator of p53 mRNA^57^. Higashi *et al*. (2002) demonstrated the induction of the p53 expression by 6-OHDA treatment and the subsequent apoptosis-related morphological changes could be prevented with antisense Wig-1 cDNA in the catecholamine-containing cells (PC12 cells)^58^. Hence, the hypothetical downregulation of Wig-1 due to the overexpression of miR-361 could be protective.

Moreover, in our miRNA-based network, amongst the TFs of miR-361, BCL6 and HIF1A transcriptionally repressed miR-361, while GATA1 activated it. Interestingly, GATA1 is a potential key regulator of the PD-linked gene α-synuclein in the blood^59^.

Furthermore, in our study, miR-885, which is involved in the interference with cell cycle progression and cell survival and promotes cellular senescence and apoptosis in neuroblastoma cell lines^16^, demonstrated a significant overexpression in both early and advanced stage PD patients, as compared to their control counterparts. Based on our miRNA-based network, miR-885 targets IGF1R, which is involved in the cellular internalization of IGF-1 after binding to its receptor and activation of the downstream signaling pathways, such as PI3K/Akt/ GSK-3β signaling cascade^60^. miR-885 also targets CTNNB1, a key regulatory protein of canonical Wnt signaling, which regulates neurogenesis and neuronal survival. A significant reduction of the CTNNB1 protein expression in midbrain dopaminergic (DA) neurons of PD has been reported^61^. Its expression might be modified by the miR-885 downregulation. Another target of miR-885 is MAN1C1, which is involved in N-glycosylation, determining the proteins fate through mediating a role in the protein folding as well as in the misfolded protein degradation^62^. A significant down-regulation in MAN1C1 expression has been shown in the PD blood samples^63^. MAN1C1 deregulation affects N-glycosylation, which may lead to the ER stress. Hence, a hypothetical downregulation of miR-885 is likely to modify the MAN1C1 expression.

Moreover, OXR1 is a vital protein that controls cellular sensitivity to oxidative stress; as well, it is a potential neuroprotective factor in the neurodegenerative diseases^64^. OXR1 is a common target of miR-885-5p and miR-361-5p. Therefore, OXR1 overexpression due to the modification of these two miRNAs could result in the less susceptibility to the exogenous stress.

Overall, our results suggest that PBMCs miR-885-5p, miR-361-5p and miR-17-5p order their combination, can discriminate the PD samples from the controls. Furthermore, the significantly obtained biological process and the pathways of the miRNA-based network were reflective of the known processes and pathways in PD, such as the regulation of the G1/S transition of the mitotic cell cycle, the regulation of autophagy, cell differentiation, p53 pathway, the oxidative stress response, the interferon-gamma signaling pathway, and the JAK/STAT signaling pathway. Nevertheless, some limitations should be noted. First, we had limited access to subjects with the advanced stages of PD that referred to the outpatient clinics; this was probably since most of them were severely disabled to ambulate or their disease was complicated by other medical issues. Second, we were unable to include confounding factors such as PD-related medication due to the lack of access to de novo PD patients. Therefore, a larger sample size-based expression study would be needed to confirm the miRNA expression changes observed during the PD progression in this study and define the involvement of the PD-related medication in the observed differential expression of miRNAs.

Despite the preliminary character of our study, we have presented a novel candidate set of aging and cellular senescence-related miRNAs; also, a consensus-based strategy is proposed to interpret the miRNAs expression data in PD; this could contribute to the identification of the possible triggering age-related signatures in PD.

## Conclusions

Overall, we revealed 3 candidate biomarkers related to aging and cellular senescence for the first time in the patients with PD; these included miR-885-5p, miR-361-5p and miR-17-5p. Also, we presented the molecular interactions of these miRNAs in two regulatory layers, thereby clarifying the potential biochemical mechanisms underlying PD. Our preliminary data provided a rationale to further clarify the role of these miRNAs in aging and PD development through their promising candidate target genes and TFs to validate their diagnostic potential.

## Supplementary information

SI

## Data Availability

The datasets used and/or analyzed during the current study are available from the corresponding author on a reasonable request.
