# Supplementary material for: Expression Profiling of Blood microRNAs 885, 361, and 17 in the Patients with the Parkinson’s disease: Integrating Interaction Data to Uncover the Possible Triggering Age-Related Mechanisms
Source: Sci Rep. 2019 Sep 24;9:13759. doi: 10.1038/s41598-019-50256-3 (PMC6760236; doi:10.1038/s41598-019-50256-3)
Supplement: Supplementary file 1 — SI [file 41598_2019_50256_MOESM1_ESM.docx]

**Running title:** Aging and cellular senescence-related microRNAs in the Parkinson’s disease

**Manuscript title:** Expression Profiling of Blood microRNAs 885, 361, and 17 in the Patients with the Parkinson’s disease: Integrating Interaction Data to Uncover the Possible Triggering Age-Related Mechanisms

Molood Behbahanipour^1^ (MSc.), Maryam Peymani^±2,3^ (PhD.), Mehri Salari^4^ (MD.), Motahare-Sadat Hashemi^3^ (PhD.), Mohammad Hossein Nasr-Esfahani^3±^ (PhD.), Kamran Ghaedi^1,3‡±^ (PhD.)

1. Department of Biology, School of Sciences, University of Isfahan, Isfahan, Iran.
2. Department of Biology, Faculty of Basic Sciences, Shahrekord Branch, Islamic Azad

University, Shahrekord, Iran.

1. Department of Cellular Biotechnology, Cell Science Research Center, Royan Institute for Biotechnology, ACECR, Isfahan, Iran.
2. Functional Neurosurgery Research Center, Shohada Tajrish Neurosurgical Center of Excellence, Shahid Beheshti University of Medical Sciences, Tehran, Iran

Corresponding authors at:

‡Division of Cellular and Molecular Biology, Department of Biology, Faculty of Sciences, University of Isfahan, Hezar Jerib Ave., Azadi Sq., P.O. Code 81746-73441, Isfahan, Iran. Phone no: +98-31-37932479; Fax no: +98-31-37932456. kamranghaedi@sci.ui.ac.ir (K. Ghaedi),

± Corresponding authors at: Department of Cellular Biotechnology, Cell Science Research Center, Royan Institute for Biotechnology, ACECR, Royan St., Salman St., P.O. Code 816513-1378, Khorsagan, Isfahan, Iran. Phone no: +98-31-95015694; Fax no: +98-31-95015687. E-mail addresses: m.peymani@ iaushk.ac.ir (M. Peymani), kamranghaedi@sci.ui.ac.ir (K. Ghaedi), & mh.nasr-esfahani@royaninstitute.org (M.H. Nasr-Esfahani).


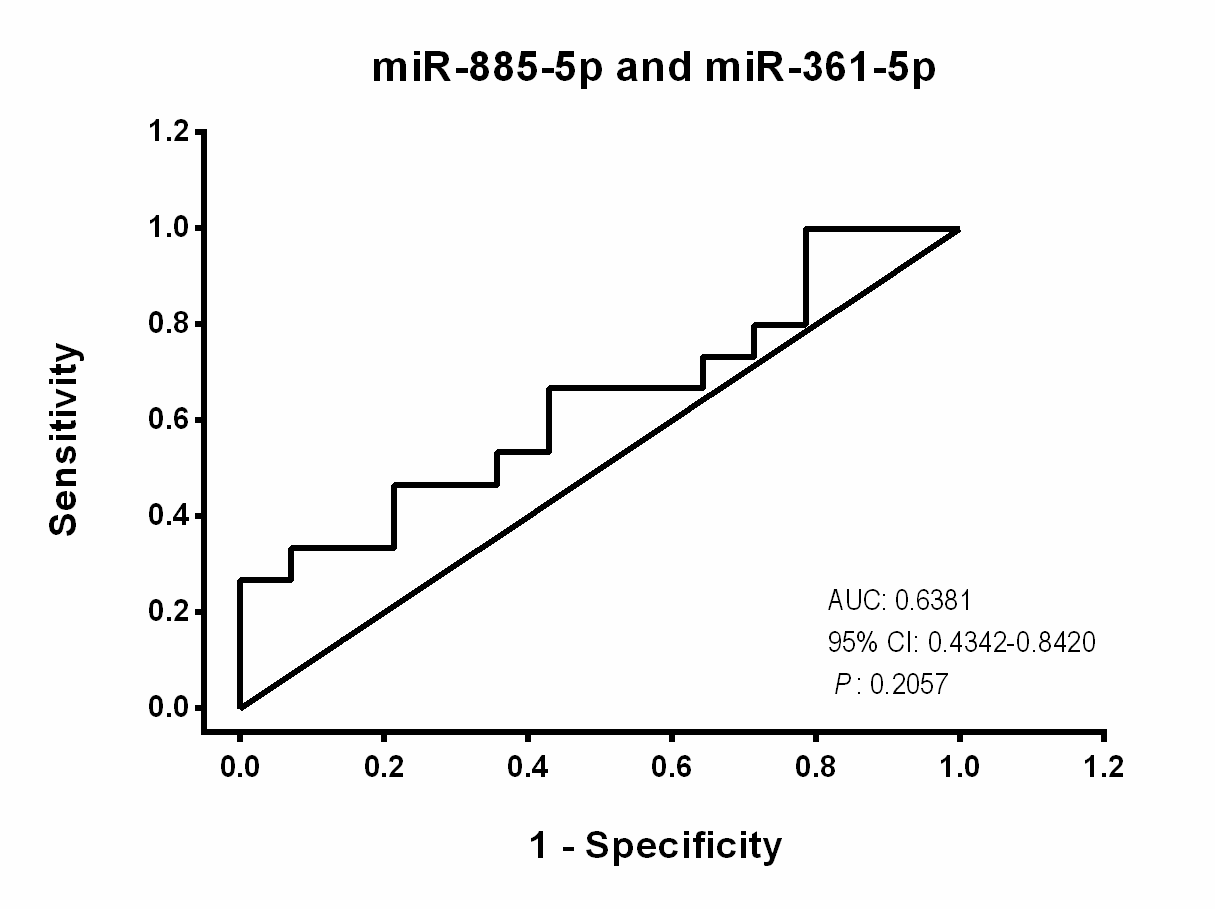


**Supplemental Figure 1** (**Figure S1).** ROC analysis using binomial logistic regression to combine miR-885-5p and miR-361-5p for the PD patients versus the healthy controls. As indicated, the AUC value was not satisfactory and significant to consider.
